# Supplementary material for: So far, so good… Similar fitness consequences and overall energetic costs for short and long-distance migrants in a seabird
Source: PLoS One. 2020 Mar 16;15(3):e0230262. doi: 10.1371/journal.pone.0230262 (PMC7075593; doi:10.1371/journal.pone.0230262)
Supplement: Attachment — Submitted filename: Pelletieretal_Response to Reviewers.pdf [file pone.0230262.s002.pdf]

RESPONSE TO REVIEWERS

JOURNAL REQUIREMENTS

REQUIREMENTS	PERSONS IN CHARGE	DONE (Y/N)	REMARKS AND/OR ANSWERS
1. Please ensure that your manuscript meets PLOS ONE's style requirements, including those for file naming (http://www.journals.plos.org/plosone/s/file?id=wjVg/PLOSOne_formatting_sample_main_body.pdf and http://www.journals.plos.org/plosone/s/file?id=ba62/PLOSOne_formatting_sample_title_authors_affiliations.pdf)	David	Y	Done
2. **We note that you have stated that you will provide repository information for your data at acceptance.** Should your manuscript be accepted for publication, we will hold it until you provide the relevant accession numbers or DOIs necessary to access your data (...).	David	Y	We shared our dataset for every figures and tables in supporting information: S1_Dataset.xlsx
3. **We note that Figure 2 in your submission contain [map/satellite] images which may be copyrighted.** All PLOS content is published under the Creative Commons Attribution License (CC BY 4.0), which means that the manuscript, images, and Supporting Information files will be freely available online, and any third party is permitted to access, download, copy, distribute, and use these materials in any way, even commercially, with proper attribution. For these reasons, we cannot publish previously copyrighted maps or satellite images created using proprietary data, such as Google software (Google Maps, Street View, and Earth). For more information, see our copyright guidelines: http://journals.plos.org/plosone/s/licenses-and-copyright. We require you to either (1) present written permission from the copyright holder to publish these figures specifically under the CC BY 4.0 license, or (2) remove the figures from your submission.	David	Y	We add this (in text and legend): "The map was generated in R (R Core Team, 2016) using the ggmap (Khale and Wickham, 2013) and ggplot2 packages (Wickham, 2016) with map tiles by Stamen Design (www.stamen.com) and data by OpenStreetMap, under ODbL, under CC BY 3.0 (creativecommons.org/licenses/by/3.0/)" ?<br><br>I sent an email with my request to the editor (2020-01-12) and I verified with Stamen Design.

RESPONSE TO REVIEWERS

REVIEWER 1

COMMENTS	PERSONS IN CHARGE	DONE (Y/N)	REMARKS AND/OR ANSWERS
**GENERAL**			
This is a well presented and considered study. A good opening paragraph introduces the evolutionary trade-offs, and this follows throughout the paper. Results are well presented in figures and tables. The trade-off between additional flight costs to reach a more distant wintering site where thermoregulatory costs are lower makes sense, and so this study offers useful evidence.	n/a	n/a	
The discussion is quite long. It includes valuable insight, however some general text could be removed or shortened so as to focus on the novelty of this study. For example, a brief further discussion could explore why these migration strategies persevere given the lack of carry-over effects in this population. Could it be to avoid competition at wintering sites, or because of familiarity with a location?	David<br>Magella	Y	We improved the Discussion.
The methods are generally well presented. Flight costs are estimated using measurements of mass and wing size recorded in the field. By my understanding from the paper, this is calculated using average measurements from all birds (please clarify). Would it have been possible to calculate this for each individual, and if so would you expect much variation because of size differences between individuals? Perhaps worth clarifying in the text either way.	David	Y	We clarified the text, in Materials and Methods/Data analysis/Energetics.
Please check grammar throughout. Some errors detailed below but there are others in text.	All	Y	Done
**SPECIFIC:**			
Title: I wonder whether the “So far, so good…” part of the title detracts from the clear and concise message in the rest of it. This is perhaps personal preference, though.	All	Y	We keep it. This is a personal choice.
Line 24-25: Consider grammar of sentence. Is there a missing word? “Allows us …” perhaps? Or, “The study of differential migration offers unique opportunities to identify such mechanisms and compare the costs and benefits of migration.”	David	Y	Corrected
Line 35-36: This was not a significant result, consider removing as the abstract provides good evidence without it.	David	Y	No, we keep this sentence. We precise that is a trend in the text.
Line 69: Grammar: change ‘stays’ to ‘stay’	David	Y	Corrected

Line 138: The list of models here could benefit from addition introduction, including, for example, the description of parameters given in Table 2. The different models could be linked more specifically to the study hypotheses to aid readers, particularly those with less experience of survival analyses who are still interested in the broader reach of this study.	Yannick	Y	We reformulated defining the general aspect of our 10 models.
Line 159: Please clarify these components, here or in the text where model structure is introduced.	Yannick	Y	Few sentences were added to define these 4 components in the previous paragraph.
Paragraph commencing line 411: This is very broad, and the advances in tracking technology have been well discussed elsewhere. I therefore wonder whether some of this paragraph detracts from the impact and interesting discussion of the results of this study. Consider streamlining, and focussing on the novelty of this study.	David<br><br>Magella	Y	We removed the first sentence.
Line 513: here, you compare DEE from two similar seabird species during the breeding season because of the absence of non-breeding information on DEE, which makes sense. However, to obtain an estimate of DEE during the breeding period for your specific population, you could use the ‘seabird FMR calculator’ proposed by Dunn et al. 2018: https://royalsocietypublishing.org/doi/full/10.1098/rsbl.2018.0190	David	Y	OK we compared our results with this calculator and the other empirical estimates, but values for DEE obtained with different methods are very similar. We modified the text in consequence.

RESPONSE TO REVIEWERS

<u>REVIEWER 2</u>

COMMENTS	PERSONS IN CHARGE	DONE (Y/N)	REMARKS AND/OR ANSWERS
**GENERAL**			
This study aims to investigate the mechanisms underlying different migration strategies, using a differential migrant, the Northern Gannet, in the western Atlantic. The authors combine data on migration distance and phenology, thermal and locomotive energetics, survival and breeding success to identify the costs and benefits of short- and long-distance migrations. The authors found no statistically significant difference in the annual survival probability, timing of arrival at the breeding site or hatching success between birds undertaking short- or long-distance migrations. Although long-distance migrants are thought to experience higher energetic costs during migration (due to flying greater distances), reduced foraging effort and thermoregulation costs in the warmer wintering area appear to compensate for this to make overall energy expenditure equal between short- and long-distance migrants during the non-breeding period. The authors conclude that there are no apparent carry-over effects as a result of long-distance migration and that the costs and benefits of different migration strategies are equal for this species.	n/a	n/a	
The study largely follows that by Garthe et al. (2012), which is a cost-benefit analysis of different migration strategies of Northern Gannets in the eastern Atlantic with similar findings. The authors have used a multi-faceted approach to present a convincing case for their conclusions and the study is an interesting and useful addition to the literature but I think there are a few things that need addressing before publication.	n/a	n/a	
**MAJOR COMMENTS:**			
1. The authors tested six different models in the survival analysis (L138), but some apparently important models have been excluded. Survival has been modelled in relation to migration strategy, year and their interaction, but none of the models test whether survival is constant across all groups and years (i.e. . ., . t) or whether there is an additive effect of migration strategy and year (i.e. g+t ., g+t t).	Yannick	Y	The 4 models were added.The selected model is still the same (phi(t) p(t)). There are a new table 2 and a new Fig. 3.
2. L441: “The high survival rate for the first 3 years of the monitoring program is because we only included birds fitted with GLS that were re-captured.” This is confusing. Does this mean that individuals were only included in the initial marked and released cohort if they were later recaptured? If so, this 100% ‘survival’ rate is meaningless and these years should be removed from the analysis.	Yannick	Y	We deleted three sentences to be clearer.

3. The m-array from the mark-recapture analysis should be made available in the supporting information so that the number of birds in each cohort is clear.	Yannick	Y	We shared our dataset for every figures and tables in supporting information (S1_Dataset.xlsx) and we added the m-array in a sheet.
4. L483: "The difference observed in mean departure date between short and long-distance migration gannets could be explained with a lower fledging success for GM migrants. Unfortunately, because we were absent at the end of breeding period in the colony, we cannot know the exact fate of each chick. However, we can argue that this difference is unrelated with migration strategy and winter destination, but more associated with variations of local resources during breeding." If the difference in departure dates is a result of lower fledging success for GM migrants, could this not be a carry-over effect? All birds are at the same colony so presumably have access to the same local resources, so a difference in fledging success between birds with different migration strategies may well be linked with those differences in migration, or to another factor that influences both migration strategy and resource availability/foraging success during breeding. To say that there is no effect on breeding success and no carry-over effects appears to be overstating the results.	David<br><br>Magella	Y	We modified the text.
5. L182: How did laying success of different migrant types compare? Were some individuals excluded from the analysis of breeding success because they did not lay eggs (i.e. failed to breed)?	David<br><br>Magella	Y	Only chick-rearing adults were instrumented with GLS. Thus, all these birds had laid eggs.
6. The manuscript requires extensive additional proof-reading/copy-editing to improve readability and correct grammatical and typographical errors throughout. E.g. L24: "…and allows to compare the costs and benefits of migration…" should be reworded as, e.g. "…and allows comparison of the costs and benefits of migration…"	Richard<br><br>Stephan	Y	Done
**MINOR COMMENTS:**			
1. L32: "Despite similar fitness consequences, we found, as expected, that the overall energetic cost of migration is higher for long-distance migrants, although the daily cost was similar between strategies. In contrast, daily maintenance and thermoregulation costs were lower for GM migrants…" This is confusing and the second sentence seems to contradict the first.	David	Y	We improved the sentence.
2. L35: "…where sea-surface temperature was 4-7°C warmer." Warmer where? In the wintering area?	David	Y	We improved the sentence.

3. L60: "Differential migration, broadly defined as observed variation in distance and destination…" Distance and destination? Or distance and timing?	David	Y	Corrected
4. L62: This sentence is unclear.	David	Y	We improved the sentence.
5. L64: "However, according to the review of Cristol et al., studies are ambiguous…" Are individual studies ambiguous? Or is it that relationships are difficult to untangle from the literature as a whole?	David	Y	We removed this sentence.
6. L77: I like the phrase "ballistic dispersal" but "dispersive migration" is perhaps more commonly used!	David	Y	Corrected
7. L92: "…that there is no long-term cost to migrate further south when winter areas provide benefits." This is slightly confusing. The authors need to specify that the "winter areas" referred to are those further from the colony. As the sentence is referring to a "basic principle of differential migration", the word "south" could be removed to make it more general.	David	Y	We removed "south"
8. L101: "Annually, around 180 nests…" This sentence seems irrelevant and misleading if these nests are not all included in the current study. From my understanding, 58 individuals were monitored for this study – those birds from which GLS devices were successfully retrieved and provided sufficient data.	David	Y	We improved this sentence.
9. L132: "We tested for permanent effect (heterogeneity in survival probability – transient effect) and temporary effect (heterogeneity in capture probability – trap-dependence)." I would suggest referring to these directly as transience and trap dependence instead of permanent and temporary effects as these terms are unclear. However, transience is not "heterogeneity in survival probability" and trap-dependence relates to heterogeneity in recapture probability. These definitions need rewriting.	Yannick	Y	We reformulated.
10. L135: Provide test results as well as p-values here.	Yannick	Y	Done.
11. L137: Define the term ˆ and state the ˆ value.	Yannick	Y	Done. c-hat was defined and the value was added.
12. L137: Provide a reference for the use of AICc.	Yannick	Y	Done (Burnham and Anderson 2002)
13. L140: "The model was built to test the difference in survival among the three categories of migrants." This sentence is confusing and could be deleted.	Yannick	Y	Deleted.
14. L158: "The CJS model is obtained…" The goodness of fit or χ2 value of the CJS model is obtained, rather than the model itself.	Yannick	Y	Done.

15. L163: …"the time spent in transit to and from the wintering area as the « fall migration season »…" Surely the fall and spring migration periods?	David	Y	Done.
16. L192: What was the elevation angle that was used?	David	Y	OK we added this information.
17. L199: What bandwidth (h-value) was used and why?	David	Y	We used a likelihood cross-validation (CV) algorithm to calculate a different bandwidth (h-value) per individual. Unlike least-squares CV that is based on minimizing the integrated squared error, likelihood CV is based on minimizing the Kullback-Leibler distance between the true underlying and the estimated distribution. Likelihood CV generally outperformed least-squared CV and is especially beneficial at sample sizes < 50 (Horne & Garton, 2006).
18. L219: This sentence is unclear.	David	Y	We improved the sentence.
19. L272: What is number of flights? This seems irrelevant to the current study.	David<br><br>Magella	N	We do not think so as it gives the perspective of all data processed in our study.
20. L288: Specify that this refers to annual survival rates for birds using different wintering areas, rather than survival on the wintering areas. We already know that these survival rates are fairly similar because migration strategy was not included in the top model of survival.	Yannick	Y	We reformulated.
21. L345: "There was no significant difference between duration of the fall and spring migrations for all migrants." Is this referring to a lack of difference between duration of fall and spring migrations within each individual? If so, these should be identical because the duration of spring migration was assumed to be the same as duration of fall migration (see L210). If not, this sentence is unclear.	David	Y	Yes this is referring to a lack of difference between duration of fall and spring migrations within each individual.
22. L380: "…was lower by 30% than during the migration seasons." Lower when? During the winter period?	David	Y	Done.

23. L383: State the effect size here.	David	N	We did not calculate the effect size with the mixed models, but we modified the sentence.
24. L476: Specifying this study's mean dates alongside those of Fifield et al. would aide comparison here.	David	N	We do not understand the comment, we are already comparing our results with those of Fifield et al.
25. L531: Preservation in what way?	David	Y	We improved the sentence.
26. L514: Define "DLW".	David	Y	Done.
27. References need proof-reading and formatting.	David	Y	Done.
28. The study species could be included in the manuscript title, although I appreciate that this may be a personal preference.	All	N	No, we prefer our title.
29. The authors may wish to consider the following additional literature:<br><br>Amélineau, F., Péron, C., Lescroël, A. et al. (2014) Windscape and tortuosity shape the flight costs of northern gannets. Journal of Experimental Biology 217: 876-885, doi: 10.1242/jeb.097915<br><br>Deakin, Z., Hamer, K., Sherley, R. et al. (2019) Sex differences in migration and demography of a wide-ranging seabird, the northern gannet. Marine Ecology Progress Series 622: 191-201, doi: 10.3354/meps12986	David	Y	We have read and added Deakin et al. (2019) in Discussion, but, although this is an interesting article, we did not add Amelineau et al., (2014) in our manuscript.
